# Differential modulation of nicotine-induced gemcitabine resistance by GABA receptor agonists in pancreatic cancer cell xenografts and in vitro

**DOI:** 10.1186/1471-2407-14-725

**Published:** 2014-09-27

**Authors:** Jheelam Banerjee, Hussein AN Al-Wadei, Mohammed H Al-Wadei, Koami Dagnon, Hildegard M Schuller

**Affiliations:** Experimental Oncology Laboratory, Department of Biomedical & Diagnostic Sciences, College of Veterinary Medicine, University of Tennessee, Knoxville, TN USA; Department of Preventive Medicine, Sana’a University, Sana’a, Yemen

## Abstract

**Background:**

Pancreatic cancer is frequently resistant to cancer therapeutics. Smoking and alcoholism are risk factors and pancreatic cancer patients often undergo nicotine replacement therapy (NRT) and treatment for alcohol dependence. Based on our report that low dose nicotine within the range of NRT causes gemcitabine resistance in pancreatic cancer, our current study has tested the hypothesis that GABA or the selective GABA-B-R agonist baclofen used to treat alcohol dependence reverse nicotine-induced gemcitabine resistance in pancreatic cancer.

**Methods:**

Using mouse xenografts from the gemcitabine--sensitive pancreatic cancer cell line BXPC-3, we tested the effects of GABA and baclofen on nicotine-induced gemcitabine resistance. The levels of cAMP, p-SRC, p-ERK, p-AKT, p-CREB and cleaved caspase-3 in xenograft tissues were determined by ELISA assays. Expression of the two GABA-B receptors, metalloproteinase-2 and 9 and EGR-1 in xenograft tissues was monitored by Western blotting. Mechanistic studies were conducted in vitro, using cell lines BXPC-3 and PANC-1 and included analyses of cAMP production by ELISA assay and Western blots to determine protein expression of GABA-B receptors, metalloproteinase-2 and 9 and EGR-1.

**Results:**

Our data show that GABA was as effective as gemcitabine and significantly reversed gemcitabine resistance induced by low dose nicotine in xenografts whereas baclofen did not. These effects of GABA were accompanied by decreases in cAMP, p-CREB, p-AKT, p-Src, p-ERK metalloproteinases-9 and -2 and EGR-1 and increases in cleaved caspase-3 in xenografts whereas baclofen had the opposite effects. In vitro exposure of cells to single doses or seven days of nicotine induced the protein expression of MMP-2, MMP-9 and EGR-1 and these responses were blocked by GABA. Baclofen downregulated the protein expression of GABA-B-Rs in xenograft tissues and in cells exposed to baclofen for seven days in vitro. This response was accompanied by inversed baclofen effects from inhibition of cAMP formation after single dose exposures to stimulation of cAMP formation in cells pretreated for seven days.

**Conclusions:**

These findings suggest GABA as a promising single agent for the therapy of pancreatic cancer and to overcome nicotine-induced gemcitabine resistance whereas treatment with baclofen may increase gemcitabine resistance.

## Background

Pancreatic cancer has a mortality > 90% within one year of diagnosis due to a lack of clinical symptoms during early stage disease and poor responsiveness to current cancer therapeutics [1]. Smoking is a well documented risk factor for this malignancy [2] and nicotine replacement therapy (NRT) frequently accompanies chemotherapy. Alcoholism also increases the risk for pancreatic cancer [3]. The synthetic selective GABA-B-receptor (GABA-B-R) agonist baclofen has been recommended for the treatment of addiction by drug abuse, including nicotine and alcohol [4, 5]. Pancreatic cancer patients may thus simultaneously be exposed to baclofen and chemotherapeutics. On the other hand, GABA is an agonist for all GABA receptors (GABA-A and GABA-B receptors) and has been used as a nutritional supplement for many years due to its calming and anxiolytic effects [6].

We have previously reported that high doses of nicotine comparable to the blood nicotine levels in heavy smokers accelerated the progression of pancreatic cancer xenografts by increasing cell proliferation and that treatment of the mice with GABA in the drinking water blocked this effect via GABA-B-receptor (GABA-B-R)-mediated inhibition of cAMP-dependent pathways [7, 8]. In accord with these findings, single dose exposures of pancreatic cancer cells in vitro to GABA or baclofen also inhibited via this mechanism cell proliferation and migration induced by the beta-adrenergic receptor agonist isoproterenol [9]. GABA additionally inhibited alcohol-induced cell proliferation and migration via reduction of cAMP formation [8].

More recently, we have shown that low dose nicotine in the range of systemic nicotine levels in moderate smokers and individuals undergoing NRT failed to increase cell proliferation-mediated pancreatic cancer xenograft progression but instead induced gemcitabine resistance by modulating apoptotic pathways [10]. Gemcitabine (Gemzar) is the leading therapeutic for pancreatic cancer, albeit with poor success [1, 11]. Gemcitabine exerts cytotoxic and apoptotic effects by inhibiting ribonucleotide reductase and DNA polymerization [12].Gemcitabine resistance frequently develops and recent findings suggest that agents that reduce p-ERK expression may help to overcome this problem [13]. Having shown that GABA and baclofen each inhibit p-ERK when administered as a single dose to pancreatic cancer cells in vitro by reducing the formation of cAMP [9], our current experiments have tested the hypothesis that both agents reverse gemcitabine resistance induced by low dose nicotine in pancreatic cancer.

## Methods

### Cell lines

The human pancreatic ductal adenocarcinoma cell lines PANC-1 and BXPC-3 were purchased from the American Type Culture Collection (Manassas, VA) and maintained in an atmosphere of 5% CO_2_ at 37°C in the culture medium recommended by the vendor. PANC-1 harbors an activating point mutation in codon 12 of the k-*ras* gene whereas BXPC-3 does not have *ras* mutations. The human PDAC cell line BXPC-3 is relatively responsive to gemcitabine whereas PANC-1 cells are relatively resistant [13]. We therefore conducted the xenograft studies in mice with BXPC-3 only while the in vitro experiments were conducted with both cell lines. Both cell lines were authenticated in June 2013 by species-specific PCR evaluation (Research Animal Diagnostic Laboratory, Columbia, MO, USA). Complete medium for PANC-1 was comprised of DMEM medium supplemented with 10% fetal bovine serum (FBS) and for BXPC-3 cells RPMI 1640 medium supplemented with 10% FBS. No antibiotics were used during maintenance of cells and in vitro experiments because antibiotics significantly alter the proteome of cells, leading to potentially aberrant signaling responses [14].

### Chemicals, reagents and antibodies

Gemcitabine (Gemzar hydrochloride) was purchased from Eli Lilly (Indianapolis, IN, USA). Baclofen, GABA and nicotine ((-)-Nicotine hydrogen tartrate) were purchased from Sigma Aldrich (St. Louis, MO, USA). Antibodies against the following proteins were purchased from Abcam (Cambridge, MA, USA): GABA-B receptor 1, GABA-B-receptor 2, beta-actin, metalloproteinase-2 (MMP-2), metalloproteinase-9 (MMP-9) and early growth response gene 1 (EGR-1). ELISA assay kits for the quantitative determination of intracellular cyclic adenosine monophosphate (cAMP) were purchased at Enzo Life Sciences (Farmingdale, NY, USA). ELISA assay kits for the quantitative determination of the phosphorylated signaling proteins extra cellular signal regulated kinase (ERK), cAMP response element binding protein (CREB), v-akt murine thymoma viral oncogene homolog (AKT), for Caspase-3 and for v-src avian sarcoma viral oncogene homolog (Src) were purchased at Invitrogen (Carlsbad, CA, USA) and MBL International Corporation (Woburn, MA, USA), respectively.

### Animal experiment

The animal experiment was approved by the University of Tennessee Knoxville Institutional Animal Care and Use Committee (IACUC). Male, 6-week-old athymic nude mice (Harlan Sprague Dawley Inc., Frederick, MD, USA) were housed in our laboratory animal facility under standard laboratory conditions with free access to food (autoclaved Purina rodent chow) and autoclaved water. All animals were subcutaneously inoculated in the flank region with BXPC-3 cells (3 × 10^6^ in 0.2 mL of PBS, viability > 95%). The mice were randomly assigned to the following treatment groups (n = 10): 1) untreated control, 2) Gemzar (Eli Lilly) twice a week intra-peritoneally (50 mg/kg), 3) Gemzar + nicotine (1 μM/L) in the drinking water with changes of water bottles twice/week, 4) GABA (10 mg/kg, 5 days a week by intra-peritoneal injection), 5) GABA + Gemzar, 6) Nicotine + GABA + Gemzar, 7) baclofen (1 mg/kg by intraperitoneal injection 5 days/week, 8) Nicotine + gemzar + baclofen. The dose of nicotine used is within the range of plasma nicotine levels in moderate smokers [15] or patients undergoing NRT [16]. The dose of GABA is within the daily dose recommended for natural GABA supplementation [6]. The dose of baclofen is within the dose recommended for the treatment of alcohol dependence [4]. Regulations by the University of Tennessee Knoxville Animal Care and Use Committee mandated that the mice be sacrificed as soon as xenografts had reached a diameter of 1.5 cm. Since xenografts from BXPC-3 cells reach that diameter within 4 weeks after subcutaneous injection, we had to start all treatments one day after injection of cancer cells in order to ensure a 4-week duration of treatments. Modulation of survival times by treatments could thus not be investigated because xenografts in the untreated control group reached that diameter 4 weeks after tumor cell inoculation and all animals were euthanized at that time to allow for comparisons among all treatment groups and controls of mechanistic data collected from xenograft tissues at an identical time point. Treatment started 1 day after subcutaneous implantation of the tumor cells. All animals were observed for 30 days after inoculation with cancer cells, while being monitored daily by a staff veterinarian board certified in Laboratory Animal Science. Body weights were recorded weekly. Water consumption was monitored by measuring the tap water with and without the nicotine in and out twice/week. Two perpendicular diameters (length and width) of each xenograft were measured with a caliper weekly, and tumor volumes were calculated as follows: (length/2) × (width^2^). At the end of the 30-day observation period, the animals were euthanized by CO_2_ inhalation. The tumors were excised and snap frozen in liquid nitrogen and stored at -80°C until analysis.

### ELISA assays

The expression of signaling proteins was determined by commercially available assay kits when such kits were available to allow for quantitative analyses of the data. In addition, cAMP levels were determined by ELISA assays. All ELISA assays were conducted following the instructions of the vendor and using an Epoch Micro plate Spectrophotometer (BioTek, Winooski, VT). Primary absorbance was read at 405 nm for cAMP and at 450 nm for p-ERK at threonine 202 and tyrosine 204, p-CREB at serine 133, p-AKT at serine 473, p-Src (630 nm reference wavelength), and at primary496 nm for cleaved caspase-3 (520 reference wavelength). All ELISA assays were conducted with 5 samples per treatment group.

### Western blots

Western blots in triplicate were used to determine the expression of proteins for which quantitative ELISA assays were not available. The protein expression of the metalloproteinases MMP-2 and MMP-9, the early growth response gene 1 (EGR-1) in xenograft tissues was thus monitored by quantitative densitometry of the western blots using previously published procedures [17].

### In vitro experiments

To obtain mechanistic insight into the observed differential effects of baclofen and GABA on xenograft growth in mice, in-vitro experiments with PANC-1 and BXPC-3 cells were conducted.

### Effects of chronic GABA and baclofen on GABA-B-R protein

Cells were seeded into 6-well plates (n = 5) at a density of 50,000 cells per well. Each of the cell lines was treated for 7 days with 10 μM, 30 μM and 50 μM of baclofen or GABA with daily replacement of drugs. Protein was isolated using standard protocol. Protein concentrations were determined using the BCA Protein Assay (Pierce, Rockford, IL) and Western blots were performed as previously described [10, 17].

### Effects of chronic baclofen on cAMP production

PANC-1 and BXPC-3 cell lines were seeded in 6-well plates at a density of 50,000 cells per well and grown in complete media (DMEM and RPMI respectively supplemented with 10% FBS). Chronic treatments with 50 μM baclofen were started on the following day and continued for 7 days with daily replenishment of drug. On day 7 the cells were switched to their respective basal media and serum starved for 24 hours. The cells were then washed with PBS and fresh basal media was added. Following exposure for 30 minutes to IBMX (1 mM) to prevent the enzymatic dissociation of intracellular cAMP, cells were first treated with baclofen (concentrations ranging from 1nM to 100 μM) for 4 hrs and then treated with 1 μM Isoproterenol for 30 min. Identical assay conditions were used in cells not pretreated with baclofen. Cells neither exposed to baclofen nor to isoproterenol served as negative controls. Cells not pretreated with baclofen and treated with isoproterenol alone for 30 minutes served as positive controls. Proteins were isolated using the manufacturer’s recommendation for the cAMP assay. Quantitative analyses of the samples (n = 5) were conducted using a cAMP ELISA kit (Enzo Life Sciences).

### Effects of nicotine and GABA on metalloproteinases and EGR-1

PANC-1 and BXPC-3 cells were seeded in 100 mm plates at a density of 200,000-300,000 cells per plate with 5 wells per treatment group (n = 5). To assess the effects of single dose exposures, cells were allowed to attach to the well surface for 24 hours and were then exposed for 4 hours to GABA (50 μM), nicotine (1 μM) or the combination of both agents. To determine the effects of chronic treatments, cells were exposed to identical concentrations of GABA, nicotine, or the combination of both for 7 days, with daily changes in culture medium containing these agents. Cells were lysed and protein expressions analyzed by triplicate Western blotting as previously described [10, 18]. Protein bands were then visualized with enhanced chemiluminescence reagent (Pierce ECL plus Western Blotting Detection Substrate). Quantitative densitometry was conducted from triplicate Western blots as previously described [17, 18].

### Statistical evaluation of data

Using GraphPad Instat 3 software (GraphPad Instant Biostatistics), the Kolmogorov and Smirnov test established that the data did not follow a Gaussian distribution and were therefore analyzed by the non-parametric Kruskal-Wallis ANOVA followed by the non-parametric Mann–Whitney test.

## Results

### Animal experiment

#### Changes in xenograft growth

**Figure 1:**
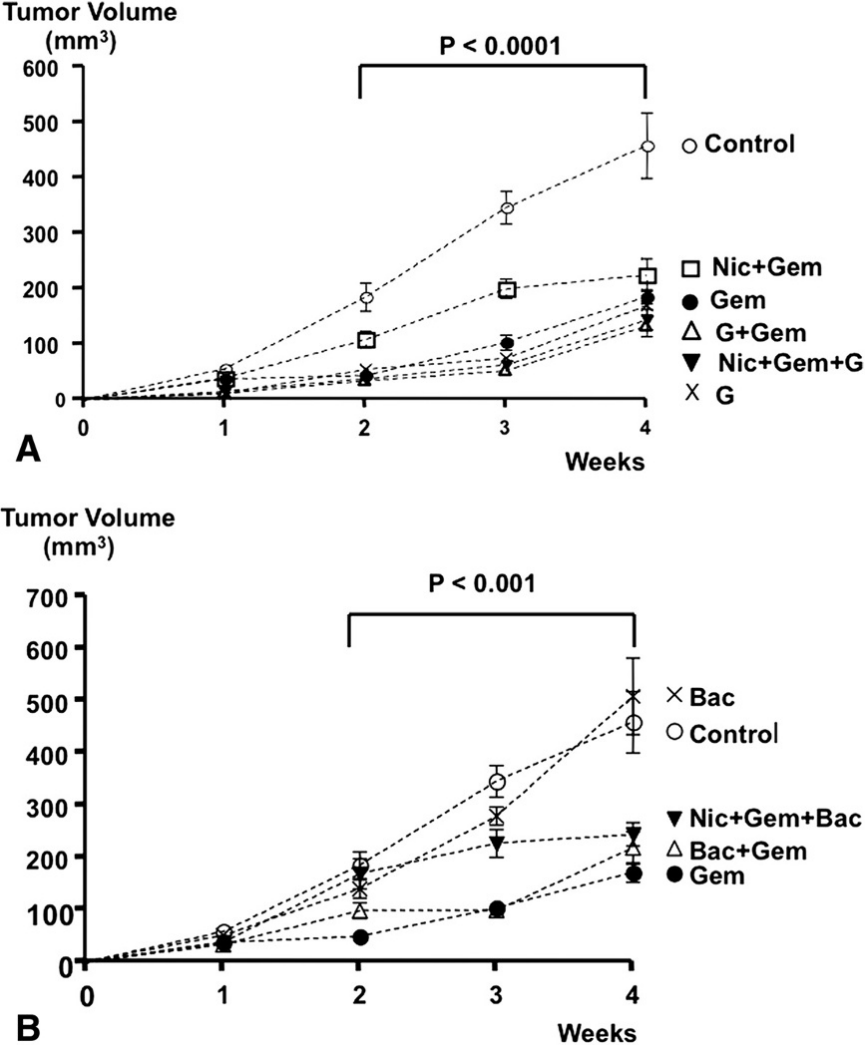
**A: Xenograft volumes.** From week 2 through 4 variations in xenograft volumes among treatment groups were significant (p < 0.0001 by Kruskal Wallis ANOVA). Gemcitabine (Gem) treatment significantly (p < 0.001 by Mann–Whitney test) reduced xenograft volumes in weeks 2 through 4 as compared with xenografts in untreated control mice. Simultaneous exposure of the animals to nicotine (Nic) in the drinking water significantly (p < 0.005 by Mann–Whitney test) reduced this effect of gemcitabine in weeks 2 and 3. Gaba (G) reversed this adverse effect of nicotine (p < 0.0005 by Mann–Whitney test) and reduced xenograft volumes below the sizes observed in animals treated with gemcitabine alone. GABA treatment alone was as effective as gemcitabine in reducing xenograft sizes. **B**: Xenograft volumes. From week 2 through 4 variations in xenograft volumes among treatment groups were significant (p < 0.001 by Kruskal Wallis ANOVA). Baclofen failed to reverse nicotine-induced gemcitabine resistance and did not significantly reduce xenograft volumes when administered as a single agent. Data points in graphs A and B are mean values and standard errors of xenograft volumes from 10 mice per treatment group.

The variation among xenograft volume medians of all treatment groups was significantly greater than expected by chance in weeks 2, 3 and 4 of the mouse experiment Figure 1A and 1B). In accord with our previous report [10], nicotine significantly increased resistance to gemcitabine in weeks 2–4 of the experiment (Figure 1A). This response was significantly reversed by simultaneous treatment of the mice with GABA (Figure 1A). By contrast, baclofen failed to significantly reduce nicotine-induced gemcitabine resistance (Figure 1B). GABA and gemcitabine cooperatively reduced xenograft volumes and GABA treatment alone was as effective as gemcitabine treatment alone (Figure 1A). By contrast, the gemcitabine-induced reduction in xenograft volumes was significantly diminished by baclofen in weeks 2 and 4 (Figure 1B), suggesting an increase in gemcitabine resistance. Moreover, treatment with baclofen alone did not significantly reduce xenograft volumes (Figure 1B).

### Modulation of cAMP levels in xenograft tissues

**Figure 2:**
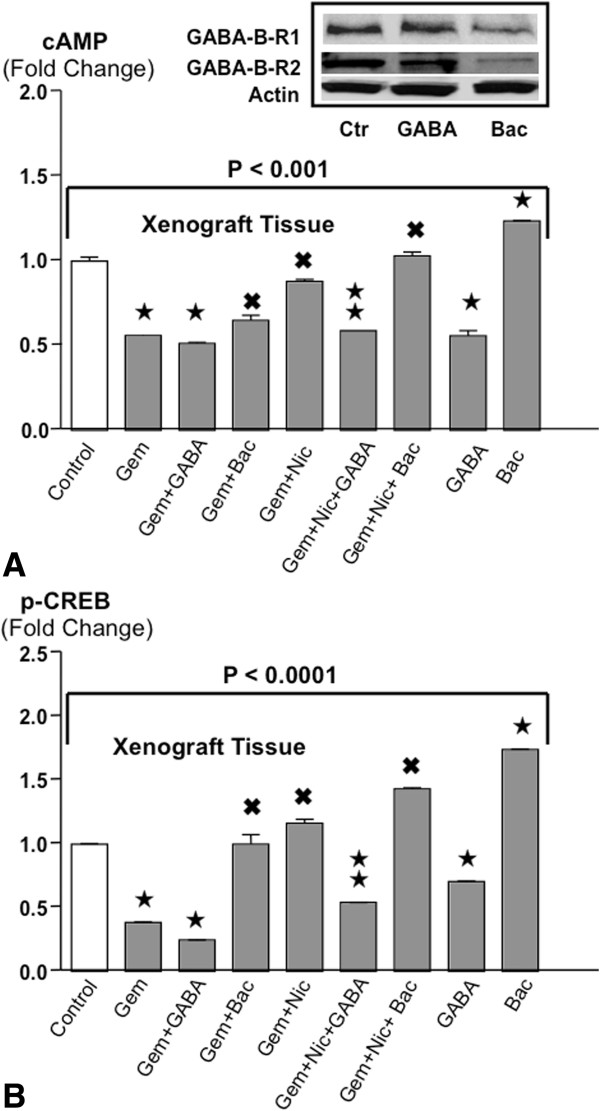
**A: Levels of cAMP in xenograft tissues.** (ELISA assays). Gemcitabine and GABA were equally effective in significantly reducing cAMP levels. Nicotine significantly reduced this effect of gemcitabine, a response reversed by simultaneous treatment with GABA. Baclofen alone significantly increased cAMP levels, diminished the effects of gemcitabine and enhanced the adverse effects of nicotine in gemcitabine treated animals. The differences among treatment groups were significant (P < 0.001 by Kruskal-Wallis ANOVA). Data are mean values and standard deviations of five randomly selected xenografts per treatment group. ★Significantly ( p < 0.01 by Mann–Whitney test) different from controls. ^✖^Significantly (p < 0.01 by Mann–Whitney test) different from gemcitabine alone.  Significantly (p < 0.01 by Mann–Whitney test) different from treatment with gemcitabine plus nicotine. The inset shows representative Western blots from xenografts exemplifying downregulated receptor proteins of both GABA-B-Rs in the mice treated with baclofen alone while receptor proteins remained unchanged in the xenograft of mice treated with GABA alone. **B**: Levels of p-CREB in xenograft tissues. ELISA assays were used to determine the levels of p-CREB in xenograft tissues. The differences among treatment groups were significant (P < 0.0001 by Kruskal-Wallis ANOVA). Gemcitabine and GABA each significantly reduced p-CREB levels, an effect significantly enhanced by the combination of both agents. Nicotine completely blocked this anti-tumorigenic effect of gemcitabine, an effect reversed by GABA. Baclofen had adverse effects similar to nicotine on gemcitabine-induced p-CREB reduction while even significantly increasing p-CREB levels over those observed in controls when given as a single agent. Data are mean values and standard deviations of five randomly selected xenografts per treatment group. ★Significantly ( p < 0.01 by Mann–Whitney test) different from controls. ^✖^Significantly (p < 0.01 by Mann–Whitney test) different from gemcitabine alone.  Significantly (p < 0.01 by Mann–Whitney test) different from treatment with gemcitabine plus nicotine.

The activation of adenylyl cyclase by G_s_-coupled receptors such as the β-ARs is the single rate-limiting step required for the intracellular formation of cyclic adenosine monophophate (cAMP). Treatment of mice with gemcitabine alone or GABA alone were equally effective in reducing cAMP production in tumor tissues whereas baclofen alone failed to inhibit cAMP formation (Figure 2A). Simultaneous treatment of the mice with gemcitabine and nicotine significantly increased cAMP levels over those observed with gemcitabine alone (Figure 2A), an effect that was reversed by GABA but not baclofen (Figure 2A).

### Changes of p-CREB levels in xenograft tissues

The transcription factor cAMP response element binding protein (CREB) is a downstream effector of cAMP that is phosphorylated by cAMP-activated protein kinase A (PKA). Gemcitabine alone significantly inhibited the phosphorylation of CREB in xenograft tissues, a response further enhanced by simultaneous treatment with GABA (Figure 2B) but not by baclofen. Nicotine completely abrogated the gemcitabine-induced reduction in p-CREB an effect significantly reversed by GABA but not baclofen (Figure 2B). GABA alone significantly decreased the levels of p-CREB whereas baclofen alone even increased p-CREB levels above control levels.

### Changes in the levels of cleaved caspase-3 in xenograft tissues

**Figure 3:**
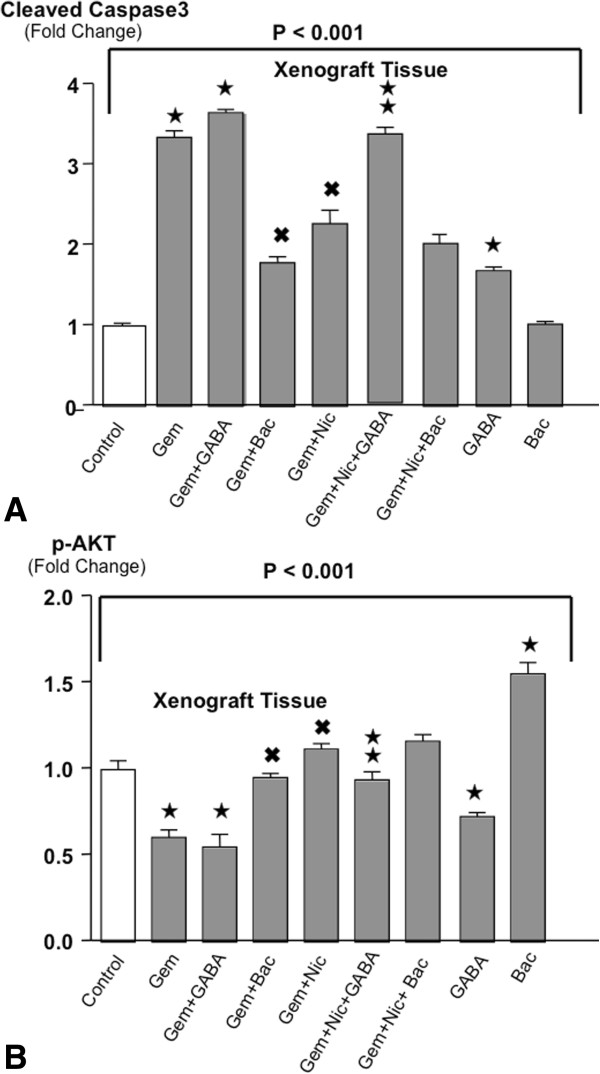
**A: Levels of cleaved caspase-3 in xenograft tissues.** Cleaved caspase-3 was measured in xenograft tissues by ELISA assay. The differences among treatment groups were significant (P < 0.001 by Kruskal-Wallis ANOVA). Gemcitabine significantly increased cleaved caspase-3 levels, a response enhanced by GABA but significantly reduced by baclofen or nicotine. GABA restored these anti-apoptotic effects of gemcitabine in nicotine treated mice while also significantly increasing cleaved caspase-3 levels when given as a single agent. Baclofen significantly reduced the apoptosis-inducing effect of gemcitabine while failing to reverse the adverse effects of nicotine. *Significantly ( p < 0.01 by Mann–Whitney test) different from controls.^✖^Significantly (p < 0.01 by Mann–Whitney test) different from gemcitabine alone.  Significantly (p < 0.01 by Mann–Whitney test) different from treatment with gemcitabine plus nicotine. **B**: Levels of p-AKT in xenograft tissues (ELISA assays). Variation in p-AKT levels among treatment groups was significant (p < 0.001 by Krsukal-Wallis ANOVA). Gemcitabine and GABA each significantly reduced p-AKT levels whereas baclofen significantly increased p-AKT levels above those observed in control mice. Nicotine and baclofen each significantly diminished the inhibitory effects of gemcitabine on p-AKT. All data are mean values and standard deviations from five randomly selected xenografts per treatment group. ★Significantly ( p < 0.01 by Mann–Whitney test) different from controls. ^✖^Significantly (p < 0.01 by Mann–Whitney test) different from gemcitabine alone.  Significantly (p < 0.01 by Mann–Whitney test) different from treatment with gemcitabine plus nicotine.

Caspase-3 is cleaved to initiate apoptosis, a mechanism activated by numerous cancer therapeutics, including gemcitabine. In accord with our previous report, and the documented apoptotic effects of gemcitabine [19], xenograft tissues in gemcitabine treated mice demonstrated more than three-fold higher levels of cleaved caspase-3 than in control animals and simultaneous treatment of the animals with nicotine significantly reduced this response (Figure 3A). GABA completely reversed this adverse effect of nicotine whereas baclofen did not. Treatment with GABA alone significantly increased cleaved caspase-3 over the levels observed in controls (Figure 3A). Baclofen alone did not significantly alter cleaved caspase-3 levels in comparison with controls but significantly reduced gemcitabine-induced induction of caspase-3 (Figure 3A).

### Modulation of p-AKT levels in xenograft tissues

The signaling protein AKT is involved in the regulation of cell proliferation and apoptosis, is activated downstream of β-ARs in pancreatic cancer cells in a cAMP-dependent manner, and AKT inhibitors are being used in combination with cancer therapeutics to improve clinical outcomes in pancreatic cancer patients. Treatment with gemcitabine or GABA alone significantly reduced the levels of p-AKT in xenograft tissues whereas baclofen alone significantly induced p-AKT (Figure 3B). Simultaneous treatment of the mice with gemcitabine and nicotine completely abrogated the inhibitory effects of gemcitabine on p-AKT. This adverse effect of nicotine was significantly reduced in animals receiving GABA (Figure 3B).

### Modulation of p-Src levels in xenograft tissues

**Figure 4:**
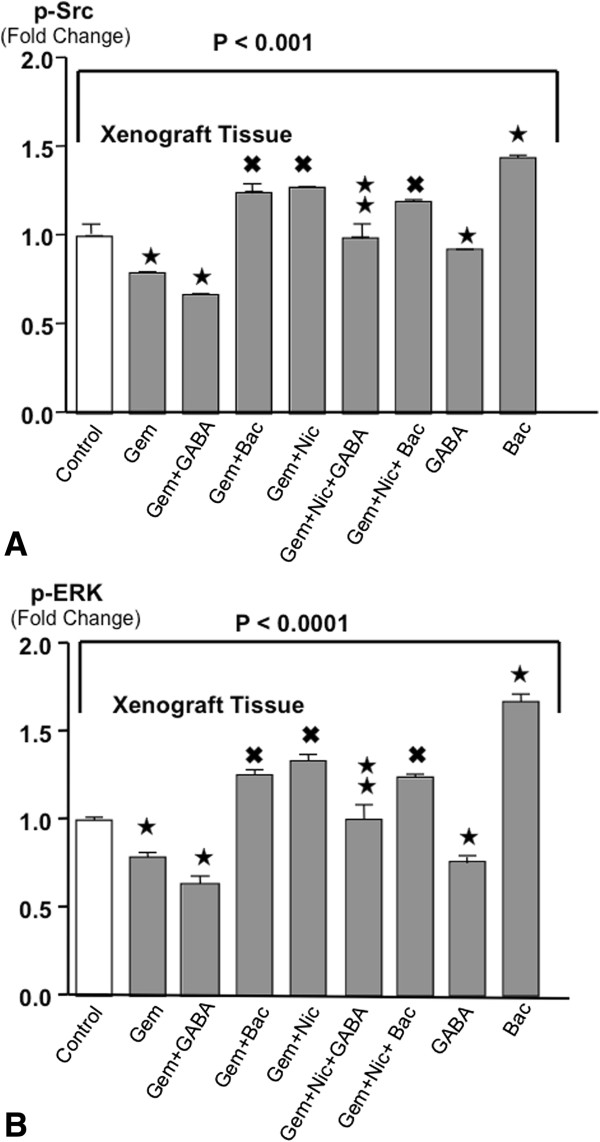
**A: Levels of p-Src in xenograft tissues (ELISA assay).** Variations of p-Src among treatment groups were significant (p < 0.001 by Kruskal-Wallis ANOVA). Gemcitabine and GABA alone and in combination significantly reduced the levels of both phosphorylated signaling proteins whereas baclofen significantly increased their levels. Baclofen and nicotine each significantly reduced the inhibitory effects of gemcitabine on both proteins. GABA significantly restored the effects of gemcitabine in the presence of nicotine. All data are mean values and standard deviations of five randomly selected xenografts per treatment group. ★Significantly ( p < 0.01 by Mann–Whitney test) different from controls. ^✖^Significantly (p < 0.01 by Mann–Whitney test) different from gemcitabine alone.  Significantly (p < 0.01 by Mann–Whitney test) different from treatment with gemcitabine plus nicotine. **B**: Levels of p-ERK in xenograft tissues (ELISA assays). Differences among treatment groups were significant (p < 0.0001 by Kruskal-Wallis ANOVA). Treatment-induced changes in p-ERK were similar to those of p-Src. Data are mean values and standard deviations of five randomly selected xenografts per treatment group. ★Significantly ( p < 0.01 by Mann–Whitney test) different from controls. ^✖^Significantly (p < 0.01 by Mann–Whitney test) different from gemcitabine alone.  Significantly (p < 0.01 by Mann–Whitney test) different from treatment with gemcitabine plus nicotine.

The signaling protein Src participates in the regulation of cell proliferation, is activated in pancreatic cancer cells in a cAMP-dependent manner, and is among the molecular targets currently utilized for the therapy of pancreatic cancer. Gemcitabine alone and GABA alone each significantly reduced p-Src in xenograft tissues (Figure 4A) and the combination treatment with both agents further enhanced this effect. By contrast, baclofen alone or in the presence of gemcitabine significantly induced p-Src above the levels observed in controls (Figure 4A) and was as effective as nicotine in abrogating the inhibitory effects of gemcitabine on Src phosphorylation. GABA significantly reduced this adverse effect of nicotine (Figure 4A).

### Changes in p-ERK levels of xenograft tissues

The signaling proteins ERK1/2 are involved in the regulation of cell proliferation. They are activated in pancreatic cancer cells downstream of the epidermal growth factor receptor (EGFR), which is activated by cAMP-dependent transactivation of the receptor itself and by cAMP-induced production of EGF. Gemcitabine and GABA each significantly reduced the levels of p-ERK in xenograft tissues when given as single agent treatments and this effect was significantly enhanced when these agents were given in combination (Figure 4B). Nicotine completely abrogated gemcitabine-induce p-ERK inhibition, increasing p-ERK levels significantly above the levels observed in controls and this effect was significantly reduced by GABA (Figure 4B). By contrast, baclofen significantly increased the levels of p-ERK in the presence and absence of gemcitabine (Figure 4B).

### Modulation in the protein expression of MMP-9, MMP-2 and EGR-1 of xenograft tissues

**Figure 5:**
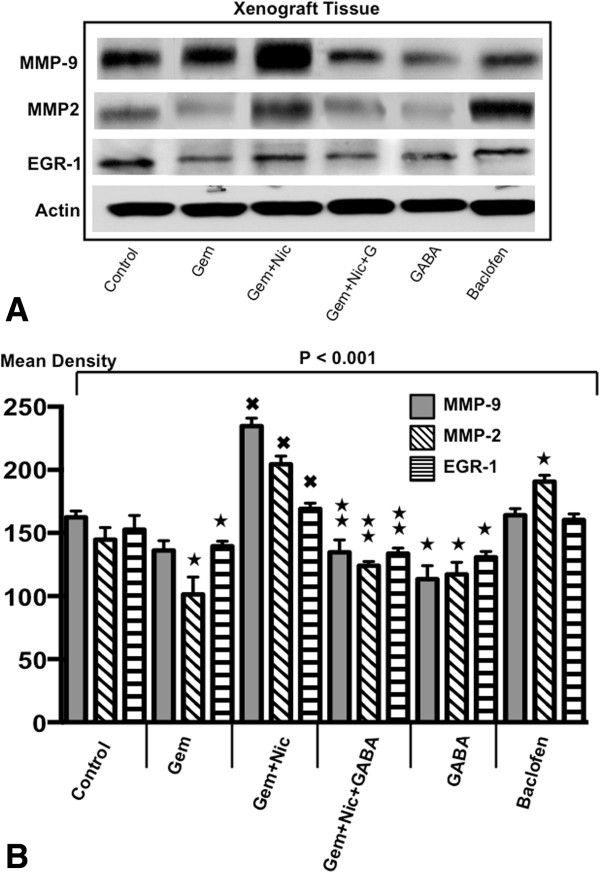
**A: Western blots from xenograft tissues.** Changes in the protein expression of metalloproteinases MMP-9 and MMP-2 and of the transcription factor EGR-1 in xenograft tissues of the six treatment groups. **B**: Densitometry data of proteins exemplified in Figure 5A. Quantitative densitometry data were generated from triplicate western blots for the determination of MMP-9, MMP-2 and EGR-1in xenograft tissues. The differences among treatment groups were significant (p < 0.001 by Kruskal-Wallis ANOVA). Gemcitabine treatment alone reduced the protein expression of MMP-2 and EGR-1. Simultaneous treatment with nicotine abrogated these responses and additionally increased MMP-9 expression above control levels. GABA showed strong inhibiting effects on all three proteins as a single agent and in combination with gemcitabine. ★Significantly ( p < 0.01 by Mann–Whitney test) different from controls. ^✖^Significantly (p < 0.01 by Mann–Whitney test) different from gemcitabine alone.  Significantly ( < 0.01 by Mann–Whitney test) different from treatment with gemcitabine plus nicotine.

The metalloproteinases MMP-2 and MMP-9 are involved in the regulation of cancer cell migration and metastasis, while the transcription factor EGR-1 participates in the regulation of cell proliferation, migration and apoptosis. Treatment with gemcitabine significantly reduced the protein expression of MMP-2 and EGR-1 in xenograft tissues while the protein expression of MMP-9 did not change significantly (Figure 5A, 5B). Nicotine significantly induced the expression of both metalloproteinases and EGR-1 above the levels of controls when administered simultaneously with gemcitabine while also significantly reversing the gemcitabine-induced inhibition of EGR-1 expression (Figure 5A, 5B). GABA reversed these effects of nicotine and GABA treatment alone reduced both metalloproteinases below control levels. Baclofen induced MMP-2 expression while having little effects on MMP-9 or EGR-1.

### In vitro studies

#### Modulation of the function and expression of GABA-B receptors by chronic baclofen

**Figure 6:**
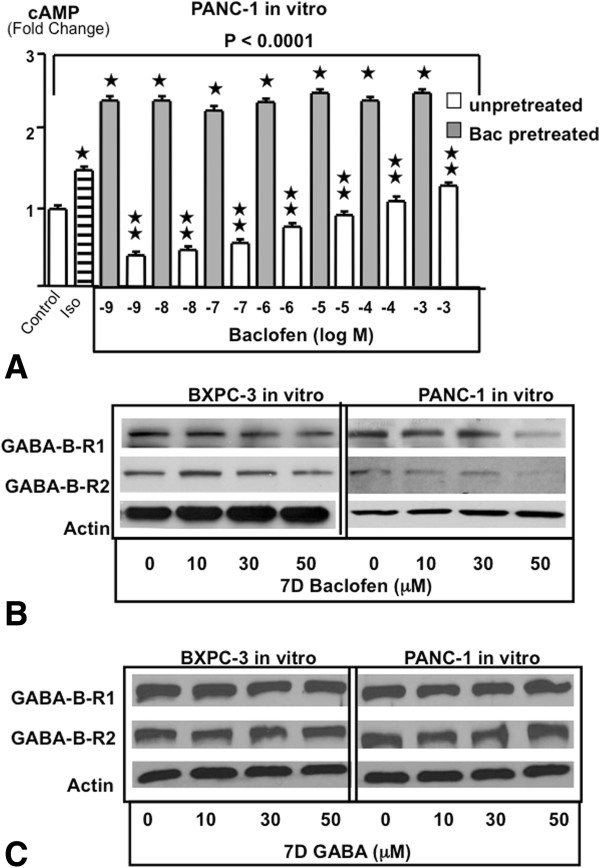
**A: Intracellular cAMP in vitro in PANC-1 cells (ELISA assay).** The differences among treatment groups were significant (p < 0.0001 by Kruskal-Wallis ANOVA). Exposure of unpretreated cells to the selective beta-adrenergic receptor agonist isoproterenol (1 μM) significantly increased cAMP levels. This response was significantly inhibited by the selective GABA-B-R agonist baclofen, with increasing concentrations yielding lesser inhibition. By contrast, none of the baclofen concentrations tested inhibited cAMP stimulation by isoproterenol in cells pretreated for seven days with baclofen (50 μM) and baclofen even significantly increased cAMP levels above those observed with isoproterenol alone. ★Significantly ( p < 0.01 by Mann–Whitney test) different from controls.  Significantly ( < 0.01 by Mann–Whitney test) different from cells pretreated for 7 days with baclofen. Data are mean values and standard deviations of five samples per treatment group. Identical assays conducted with BXPC-3 cells yielded similar results (data not shown). **B**: Western blots of GABA-B receptors in pancreatic cancer cell lines in vitro. The protein expression of the two GABA-B receptors decreased in response to ascending concentrations of chronic (7 days) baclofen in BXPC-3 and PANC-1 cell in vitro. **C**: Western blots of GABA-B receptors in BXPC-3 and PANC-1 cells in vitro. The protein expression of the two GABA-B receptors in response to ascending concentrations of chronic (7 days) GABA remained unchanged in BXPC-3 and PANC-1 cell in vitro.

The two GABA-B receptors (GABA-B-R1, GABA-B-R2) are coupled to the inhibitory G-protein G_i_ that reduces the formation of cAMP by inhibiting the activation of adenylyl cyclase by receptors such as the β-ARs, that are coupled to the stimulatory G-protein G_s_ [9]. The proliferation and migration of pancreatic cancer cells is stimulated by agonists for the G_s_-coupled beta-adrenergic receptors and nicotine triggers this response by stimulating the production of their physiological agonists norepinephrine and epinephrine in pancreatic cancer cells [9, 20]. The failure of the selective GABA-B-R agonist baclofen to inhibit xenograft growth in the mouse experiment suggested to us that chronic treatment with baclofen might have desensitized the GABA-B-Rs. This hypothesis is supported by cAMP assays conducted in BXPC-3 and PANC-1 cells in vitro and exemplified in Figure 6A. The selective beta-adrenergic agonist isoproterenol significantly increased intracellular cAMP levels and this response was reduced by single doses of baclofen at concentrations from 1 nM through 10 μM. There was an inverse dose–response relationship, with increasing concentrations being less inhibitory and no inhibition detectable at the highest dose of 100 μM baclofen (Figure 6A), a finding consistent with receptor desensitization by high agonist concentrations. Pretreatment of the cells for 7 days with 50 μM baclofen resulted in a complete reversal of GABA-B-R function as evidenced by significant stimulation of cAMP formation in response to each single baclofen concentration tested even above the levels observed with isoproterenol (Figure 6A). These findings are in accord with the results of Western blots that revealed downregulated protein expression of both GABA-B-Rs in BXPC-3 and PANC-1 cells exposed for 7 days to 50 μM baclofen (Figure 6B). By contrast, identical treatment of both cell lines with GABA, which was highly effective in inhibiting xenograft growth, did not change the protein expression of these receptors (Figure 6C).

### Effects of single dose and chronic nicotine and GABA on metalloproteinases and EGR-1 in vitro

**Figure 7:**
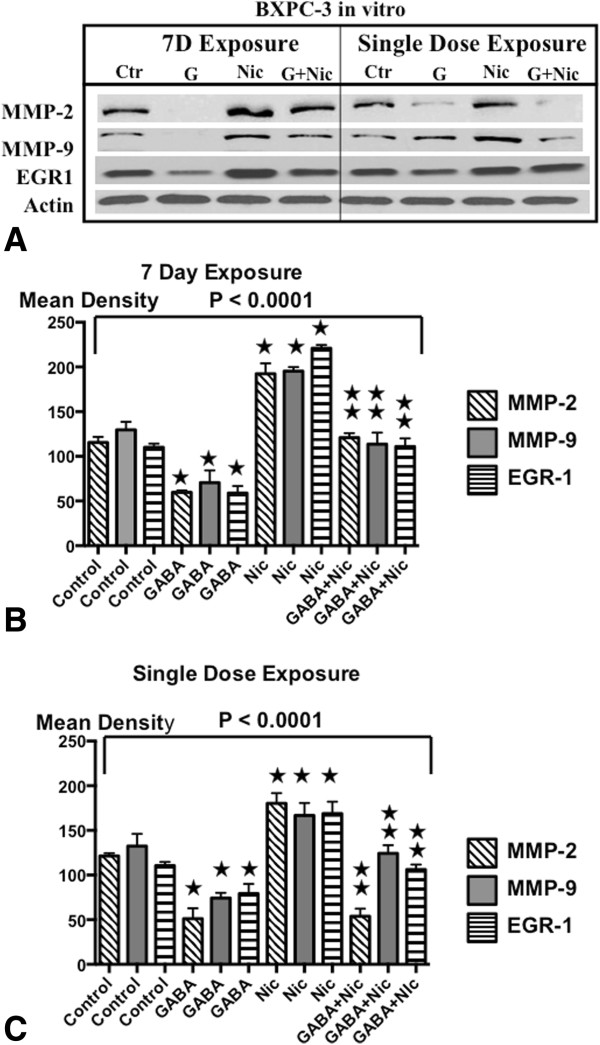
**A: Western blots of signaling proteins in BXPC-3 cells in vitro.** Changes in the protein expression of the metalloproteinases MMP-2 and MMP-9 and on the transcription factor EGR-1 exposure to single doses or 7 days of GABA (50 μM) , nicotine (1 μM) or the combination of both agents in BXPC-3 cells in vitro. **B**: Densitometry data of signaling proteins in BXPC-3 cells treated for 7 days. Quantitative densitometry was conducted from triplicate western blots of BXPC-3 cells after 7 days of treatment. The differences among treatment groups for MMP-2, MMP-9 and EGR-1 were significant (p < 0.0001 by Kruskal-Wallis ANOVA). Nicotine induced the expression of these three proteins while GABA had strong inhibiting effects in the absence and presence of nicotine. ★Significantly ( p < 0.005 by Mann–Whitney test) different from controls.  Significantly ( < 0.005 by Mann–Whitney test) different from treatment with nicotine. **C:** Densitometry data of signaling proteins in BXPC-3 cells after single dose treatments. Quantitative densitometry was conducted from triplicate western blots of BXPC-3 cells after single dose treatments treatment. The differences among treatment groups for MMP-2, MMP-9 and EGR-1 were significant (p < 0.0001 by Kruskal-Wallis ANOVA). Nicotine induced the expression of these three proteins while GABA had strong inhibiting effects in the absence and presence of nicotine. ★Significantly ( p < 0.005 by Mann–Whitney test) different from controls.  Significantly ( < 0.005 by Mann–Whitney test) different from treatment with nicotine.

**Figure 8:**
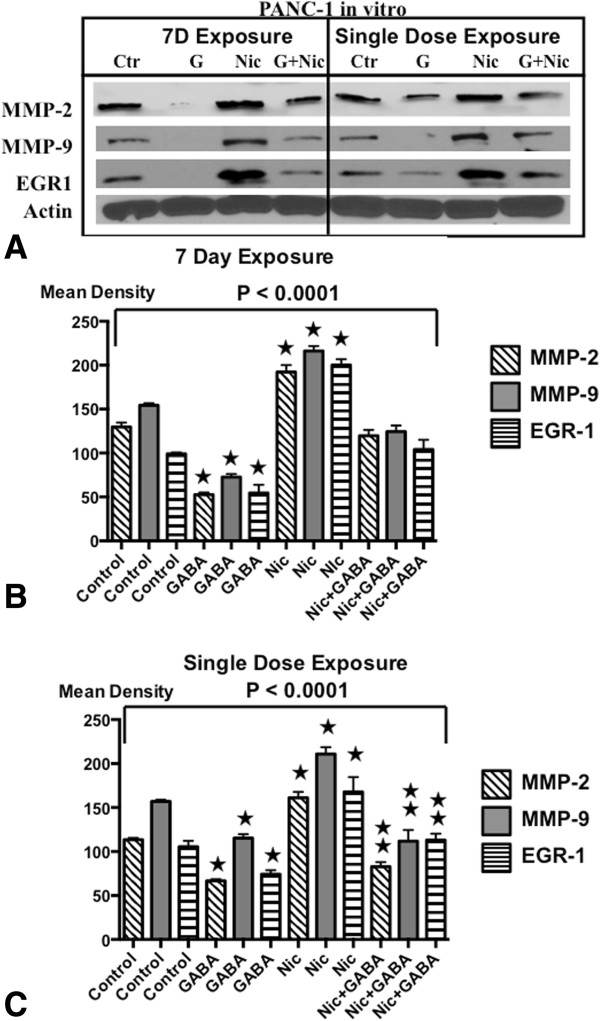
**A: Western blots of signaling proteins in PANC-1 cells in vitro.** Exposure to single doses or 7 days of GABA (50 μM), nicotine (1 μM) or the combination of both agents modulated the protein expression of the metalloproteinases MMP-2 and MMP-9 and on the transcription factor EGR-1 in PANC-1 cells in vitro. **B**: Densitometry data from PANC-1 cells treated for 7 days. Quantitative densitometry was generated from triplicate western blots of PANC-1 cells after 7 days of treatment. The differences among treatment groups for MMP-2, MMP-9 and EGR-1 were significant (p < 0.0001 by Kruskal-Wallis ANOVA). Nicotine induced the expression of these three proteins while GABA had strong inhibiting effects in the absence and presence of nicotine. ★Significantly ( p < 0.005 by Mann–Whitney test) different from controls.  Significantly ( < 0.005 by Mann–Whitney test) different from treatment with nicotine. **C**: Quantitative densitometry data after single dose treatments of PANC-1 cells. The differences among treatment groups for MMP-2, MMP-9 and EGR-1 were significant (p < 0.0001 by Kruskal-Wallis ANOVA). Nicotine induced the expression of these three proteins while GABA had strong inhibiting effects in the absence and presence of nicotine. ★Significantly ( p < 0.005 by Mann–Whitney test) different from controls.  Significantly ( < 0.005 by Mann–Whitney test) different from treatment with nicotine.

Western blots of xenograft tissues suggested that chronic nicotine induced while GABA inhibited MMP-9, MMP-2 and EGR-1 in pancreatic cancer. We therefore treated cell lines BXPC-3 and PANC-1 in vitro with a single dose or for 7 days with nicotine (1 μM), GABA (50 μM) or the combination of both agents and assessed the protein expression of these three targets by Western blots. As Figures 7A-C and 8A-C show, acute as well as chronic exposure to nicotine significantly induced the protein expression of both metalloproteinases and EGR-1 in both cell lines, responses significantly reduced by simultaneous exposure to GABA. Moreover, GABA alone reduced the protein expression of all three targets significantly below control levels, an effect particularly prominent after 7 days of exposure.

## Discussion

Our experimental in vivo and in vitro data show, for the first time, that GABA effectively reversed nicotine-induced gemcitabine resistance of pancreatic cancer. Moreover, our findings suggest that single agent therapy with GABA is as effective as gemcitabine in pancreatic cancer. The observed nicotine-induced changes in p-ERK, p-Src, p-CREB, p-AKT and caspase-3 in xenograft tissues and in vitro are in accord with reports describing mechanisms of nicotine-induced pancreatic cancer cell proliferation [18], inhibition of apoptosis [10, 21] and increase in gemcitabine resistance [21, 22]. The inhibitory effects of GABA on these targets confirm earlier reports that the activation of ERK, Src and CREB in pancreatic cancer cells is cAMP-dependent and reduced by activation of the Gi-coupled GABA-B receptors [9]. While gemcitabine is primarily known for its cytotoxic and pro-apoptotic effects, it has also been shown to act as an analgesic that decreased cAMP by reducing the degradation of Gi [23]. The gemcitabine-induced reductions in cAMP. P-CREB, p-ERK, p-Src and p-AKT are in accord with these findings. Our in vitro and in vivo findings that nicotine induced metalloproteinases 9 and 2 are in accord with analyses by PCR and immunohistochemistry that revealed increases in MMP-9 associated with induction of vascular endothelial growth factor in pancreatic cancer cells exposed in vitro to nicotine [24]. Collectively, these findings are of immediate translational relevance and warrant clinical trials with GABA alone and as a tool to overcome nicotine-induced gemcitabine resistance in pancreatic cancer patients.

Effects of nicotine on the EGR-1 gene in pancreatic cancer cells has not been studied to date. The transcription factor EGR-1participates in the regulation of cell proliferation, differentiation and apoptosis and can act as a tumor promoter or tumor suppressor pending on the type of cancer under study and the experimental conditions used [25–29]. Our current in vivo and in vitro findings are in accord with observations that nicotine induced EGR-1 expression in pheochromocyatoma cells [30] and that EGR-1 is involved in the conversion of norepinephrine to epinephrine by the enzyme phenylethanolamine N-methyltransferase [31]. We have recently shown that nicotine induces the synthesis and release of norepinephrine and epinephrine from pancreatic cancer cells with both neurotransmitters acting as autocrine growth factors via cAMP-dependent signaling cascades that were inhibited by GABA [17, 18]. The observed modulation of EGR-1 expression in the current experiments are thus likely associated with changes in the activity of these pathways.

Similar to many classic cancer therapeutics, gemcitabine is a synthetic agent that induces apoptosis and cytotoxicity not only in cancer cells but also in normal epithelial cells, resulting in adverse side effects [11, 13, 32]. By contrast, GABA is a physiological product of the mammalian organism that serves as the major inhibitory neurotransmitter in the nervous system [33]. GABA also restores cAMP homeostasis via inhibition of adenylyl cyclase activation by the G_i_-coupled GABA-B-Rs when G_s_-coupled receptors cause excessive cAMP formation in epithelial cells and the cancers derived from them [9, 17, 34–36]. Germinated brown rice [37], grapes and wine [38] are nutritional sources rich in GABA and GABA has been safely used as a nutritional supplement for many years because of its calming and anxiolytic effects [6]. Unlike therapy with synthetic GABA receptor agonists, natural GABA supplementation is virtually without side effects [6]. In light of the fact that the dose of GABA used in our animal experiment was within the range of GABA supplementation used in humans, GABA therapy can therefore be achieved by a nutritional approach without the adverse side effects typical for most classic cancer therapeutics. However, subsets of pancreatic cancers overexpress the π-subunit of the GABA-A receptor, converting the cancer inhibiting functions of GABA to cancer stimulating activity [39]. Individuals with this abnormality should therefore be excluded from any attempts to treat or prevent pancreatic cancer with GABA.

Contrary to GABA, the selective agonist for GABA-B-Rs, baclofen, failed to reduce nicotine-induced gemcitabine resistance, did not improve the efficacy of gemcitabine and did not inhibit the growth of xenografts when used as single agent treatment. In fact, baclofen alone significantly increased the levels of cAMP. p-CREB, p-AKT, p-Src and p-ERK in xenograft tissues while significantly reducing the levels of cleaved caspase-3. These findings are of concern, as they suggest that treatment of drug addictions including nicotine and alcohol with baclofen [4, 5] may promote the progression of pancreatic cancer while also increasing gemcitabine resistance. In light of the fact that habitual ingestion of liquor-strength alcohol and smoking are risk factors for pancreatic cancer [2, 3], individuals undergoing baclofen therapy for alcohol or nicotine dependence may harbor precancerous pancreatic lesions that are promoted to overt cancer by baclofen.

The downregulation of receptor proteins for both GABA-B receptors observed in xenograft tissues of mice treated with baclofen in conjunction with the decrease in xenograft cAMP levels of that treatment group suggest that the observed adverse effects of baclofen were caused by agonist-induced downregulation and desensitization of these receptors. This hypothesis is supported by our in vitro findings that exposure of BXPC-3 or PANC-1 cells for seven days to baclofen reduced the protein expression of both GABA-B-Rs in a concentration-dependent manner whereas identical exposures of the cells to GABA did not change receptor protein. Measurements of intracellular cAMP additionally showed that pretreatment of cells for seven days with baclofen completely reversed the cAMP-inhibiting effects observed after single doses of baclofen in cells not pretreated with baclofen. These findings are in accord with the paradoxical superactivation of adenylyl cyclase that has been reported in response to persistent exposure of G_i_-coupled receptors to agonist concentrations that yielded receptor saturation [40]. In turn, the resulting increases in xenograft cAMP of mice treated for 4 weeks with baclofen triggered the tumor promoting effects of this agent in the current animal experiment. Identical chronic treatments with GABA in vivo and in vitro did not trigger desensitization of GABA-B-Rs and superactivation of adenylyl cyclase because GABA binds with equal affinity to the large family of GABA-A receptors that are also expressed in pancreatic cancer cells [39]. Identical treatments with the GABA-B-R selective baclofen thus saturated the binding sites of GABA-B-Rs whereas a significant proportion of the non-selective GABA bound to GABA-A receptors, resulting in lower occupancy of GABA-B-R binding sites.

## Conclusions

Our data suggest that GABA may have significant therapeutic effects on pancreatic cancer when administered as a single agent and may reduce potential nicotine-induced gemcitabine resistance. Our data further suggest that baclofen is contra-indicated in pancreatic cancer patients because it may induce cancer progression and increase gemcitabine resistance.
